# (*E*)-2-Hydr­oxy-3-methoxy­benzaldehyde thio­semicarbazone

**DOI:** 10.1107/S1600536808014475

**Published:** 2008-05-17

**Authors:** Ren-Gao Zhao, Wei Zhang, Ji-Kun Li, Li-Ya Zhang

**Affiliations:** a Department of Materials Science and Chemical Engineering, Taishan University, 271021 Taian, Shandong, People’s Republic of China; b Feng Cheng Senior High School, 271100 Laiwu, Shandong, People’s Republic of China

## Abstract

In the title compound, C_9_H_11_N_3_O_2_S, intra­molecular O—H⋯O and N—H⋯N hydrogen bonds contribute to the planarity of the mol­ecular skeleton. Inter­molecular N—H⋯O hydrogen bonds link the mol­ecules into zigzag chains along the *b* axis; these mol­ecules are futher paired by π–π inter­actions [centroid–centroid distance 4.495 (5) Å]. The crystal structure also exhibits weak inter­molecular N—H⋯S and O—H⋯S hydrogen bonds.

## Related literature

For related crystal structures, see: Joseph *et al.* (2006 ▶). For biological activities of thio­semicarbazone Schiff bases, see: Kasuga *et al.* (2001 ▶); Fonari *et al.* (2003 ▶).
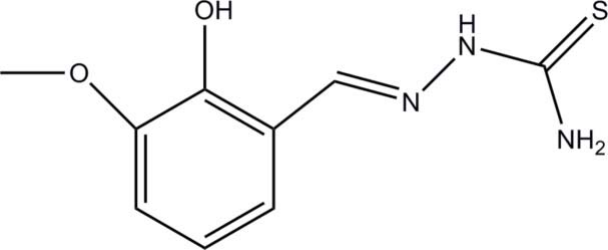


## Experimental

### 

#### Crystal data

C_9_H_11_N_3_O_2_S
*M* *_r_* = 225.27
Monoclinic, 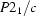

*a* = 7.057 (3) Å
*b* = 14.673 (5) Å
*c* = 10.738 (4) Å
β = 108.412 (7)°
*V* = 1055.0 (7) Å^3^
*Z* = 4
Mo *K*α radiation
μ = 0.29 mm^−1^
*T* = 273 (2) K
0.15 × 0.12 × 0.10 mm

#### Data collection

Bruker SMART CCD area-detector diffractometer
Absorption correction: multi-scan (*SADABS*; Sheldrick, 1996 ▶) *T*_min_ = 0.958, *T*_max_ = 0.972
5510 measured reflections
1872 independent reflections
1023 reflections with *I* > 2σ(*I*)
*R*_int_ = 0.071

#### Refinement

*R*[*F* ^2^ > 2σ(*F* ^2^)] = 0.059
*wR*(*F* ^2^) = 0.163
*S* = 1.10
1872 reflections
138 parameters
H-atom parameters constrained
Δρ_max_ = 0.18 e Å^−3^
Δρ_min_ = −0.28 e Å^−3^

### 

Data collection: *SMART* (Siemens, 1996 ▶); cell refinement: *SAINT* (Siemens, 1996 ▶); data reduction: *SAINT*; program(s) used to solve structure: *SHELXS97* (Sheldrick, 2008 ▶); program(s) used to refine structure: *SHELXL97* (Sheldrick, 2008 ▶); molecular graphics: *SHELXTL* (Sheldrick, 2008 ▶); software used to prepare material for publication: *SHELXTL*.

## Supplementary Material

Crystal structure: contains datablocks global, I. DOI: 10.1107/S1600536808014475/cv2411sup1.cif

Structure factors: contains datablocks I. DOI: 10.1107/S1600536808014475/cv2411Isup2.hkl

Additional supplementary materials:  crystallographic information; 3D view; checkCIF report

## Figures and Tables

**Table 1:** Hydrogen-bond geometry (Å, °)

*D*—H⋯*A*	*D*—H	H⋯*A*	*D*⋯*A*	*D*—H⋯*A*
O1—H1⋯O2	0.82	2.14	2.610 (4)	116
N3—H3*A*⋯N1	0.86	2.23	2.592 (5)	105
O1—H1⋯S1^ii^	0.82	2.69	3.290 (3)	131
N2—H2⋯S1^iii^	0.86	2.62	3.470 (4)	172
N3—H3*B*⋯O1^iv^	0.86	2.28	2.943 (4)	134

Symmetry codes: (ii) 
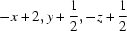
; (iii) 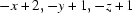; (iv) 
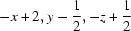
.

**Fig. 1.**
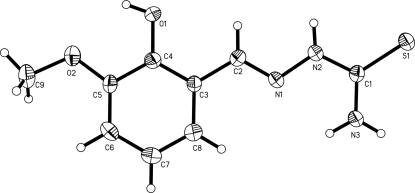
The molecular structure of (I) showing the atomic numbering and 30% probability displacement ellipsoids.

## supplementary crystallographic information

### Comment

Thiosemicarbazone Schiff-bases have been investigated in terms of their chemistry and potentially beneficial biological activities, such as antitumor, antibacterial, antiviral and antimalarial activities (Kasuga *et al.*, 2001; Fonari *et al.*, 2003). In continuation of our studies on thiosemicarbazone Schiff-bases, we report the synthesis and crystal structure of the title compound, (I).

In (I) (Fig. 1), all bond lengths and angles are normal and in a good agreement with those found in the literature (Joseph *et al.*, 2006). The intramolecular O—H···O and N—H···N hydrogen bonds (Table 2) contribute to the planarity of molecular skeleton. The intermolecular N—H···O hydrogen bonds (Table 2) link the molecules into zigzag chains along *b* axis, which are futher paired by π···π interactions proved by short intermolecular C···C distances (Table 1). The crystal packing exhibits also weak intermolecular N—H···S and O—H···S hydrogen bonds (Table 2).

### Experimental

The title compound was synthesized by the reaction of 2-hydroxy-3-methoxybenzaldehyde (0.152 g, 1 mmol) and hydrazinecarbothioamide (0.091 g, 1 mmol) in ethanol solution and stirred under reflux conditions (353 K) for 6 h. When cooled to the room temperature, the solution was filtered off and after a week orange crystals suitable for X-ray diffraction study were obtained. Yield, 0.199 g, 82%. m.p. 358–360 K.

Analysis found: C 47.94, H 4.95, N 18.62%; C_9_H_11_N_3_O_2_S requires: C 47.99, H 4.92, N 18.65%.

### Refinement

The H-atoms were geometrically positioned (C-H 0.93-0.96 Å, N-H 0.86 Å, O-H 0.82 Å), and refined as riding on their parent atoms, with *U*_iso_(H) = 1.2*U*_eq_(C-aromatic and N) and *U*_iso_(H) = 1.5*U*_eq_(*C*-methyl and O).

### Crystal data

**Table:**

C_9_H_11_N_3_O_2_S	*F*(000) = 472
*M**_r_* = 225.27	*D*_x_ = 1.418 Mg m^−^^3^
Monoclinic, *P*2_1_/*c*	Mo *K*α radiation, λ = 0.71073 Å
Hall symbol: -P 2ybc	Cell parameters from 511 reflections
*a* = 7.057 (3) Å	θ = 2.4–19.8°
*b* = 14.673 (5) Å	µ = 0.29 mm^−^^1^
*c* = 10.738 (4) Å	*T* = 273 K
β = 108.412 (7)°	Block, orange
*V* = 1055.0 (7) Å^3^	0.15 × 0.12 × 0.10 mm
*Z* = 4	

### Data collection

**Table:**

Bruker SMART CCD area-detector diffractometer	1872 independent reflections
Radiation source: fine-focus sealed tube	1023 reflections with *I* > 2σ(*I*)
graphite	*R*_int_ = 0.071
φ and ω scans	θ_max_ = 25.0°, θ_min_ = 2.4°
Absorption correction: multi-scan (*SADABS*; Sheldrick, 1996)	*h* = −8→6
*T*_min_ = 0.958, *T*_max_ = 0.972	*k* = −17→17
5510 measured reflections	*l* = −9→12

### Refinement

**Table:**

Refinement on *F*^2^	Secondary atom site location: difference Fourier map
Least-squares matrix: full	Hydrogen site location: inferred from neighbouring sites
*R*[*F*^2^ > 2σ(*F*^2^)] = 0.060	H-atom parameters constrained
*wR*(*F*^2^) = 0.163	*w* = 1/[σ^2^(*F*_o_^2^) + (0.0641*P*)^2^ + 0.0089*P*] where *P* = (*F*_o_^2^ + 2*F*_c_^2^)/3
*S* = 1.10	(Δ/σ)_max_ < 0.001
1872 reflections	Δρ_max_ = 0.19 e Å^−^^3^
138 parameters	Δρ_min_ = −0.28 e Å^−^^3^
0 restraints	Extinction correction: *SHELXL97* (Sheldrick, 2008), Fc^*^=kFc[1+0.001xFc^2^λ^3^/sin(2θ)]^-1/4^
Primary atom site location: structure-invariant direct methods	Extinction coefficient: 0.005 (2)

### Special details

**Table:**

Geometry. All e.s.d.'s (except the e.s.d. in the dihedral angle between two l.s. planes) are estimated using the full covariance matrix. The cell e.s.d.'s are taken into account individually in the estimation of e.s.d.'s in distances, angles and torsion angles; correlations between e.s.d.'s in cell parameters are only used when they are defined by crystal symmetry. An approximate (isotropic) treatment of cell e.s.d.'s is used for estimating e.s.d.'s involving l.s. planes.
Refinement. Refinement of *F*^2^ against ALL reflections. The weighted *R*-factor *wR* and goodness of fit *S* are based on *F*^2^, conventional *R*-factors *R* are based on *F*, with *F* set to zero for negative *F*^2^. The threshold expression of *F*^2^ > σ(*F*^2^) is used only for calculating *R*-factors(gt) *etc*. and is not relevant to the choice of reflections for refinement. *R*-factors based on *F*^2^ are statistically about twice as large as those based on *F*, and *R*- factors based on ALL data will be even larger.

### Fractional atomic coordinates and isotropic or equivalent isotropic displacement parameters (Å^2^)

**Table:**

	*x*	*y*	*z*	*U*_iso_*/*U*_eq_	
S1	1.0171 (2)	0.35387 (8)	0.55909 (10)	0.0534 (5)	
O1	0.8111 (5)	0.65121 (18)	−0.0190 (3)	0.0600 (10)	
H1	0.7915	0.6940	−0.0706	0.090*	
O2	0.6495 (5)	0.6610 (2)	−0.2733 (3)	0.0637 (10)	
N1	0.8421 (5)	0.4185 (2)	0.1879 (3)	0.0423 (10)	
N2	0.9111 (5)	0.4247 (2)	0.3218 (3)	0.0464 (10)	
H2	0.9351	0.4771	0.3595	0.056*	
N3	0.9079 (6)	0.2710 (2)	0.3278 (3)	0.0583 (12)	
H3A	0.8692	0.2716	0.2434	0.070*	
H3B	0.9248	0.2199	0.3692	0.070*	
C1	0.9411 (7)	0.3481 (3)	0.3936 (4)	0.0425 (11)	
C2	0.8215 (7)	0.4935 (3)	0.1257 (4)	0.0442 (12)	
H2A	0.8580	0.5480	0.1712	0.053*	
C3	0.7403 (6)	0.4938 (3)	−0.0173 (4)	0.0383 (11)	
C4	0.7340 (7)	0.5738 (3)	−0.0851 (4)	0.0410 (11)	
C5	0.6465 (7)	0.5770 (3)	−0.2212 (4)	0.0443 (12)	
C6	0.5703 (8)	0.4995 (3)	−0.2877 (4)	0.0556 (14)	
H6	0.5123	0.5011	−0.3784	0.067*	
C7	0.5789 (8)	0.4183 (3)	−0.2207 (4)	0.0625 (15)	
H7	0.5275	0.3655	−0.2669	0.075*	
C8	0.6621 (7)	0.4148 (3)	−0.0874 (4)	0.0552 (14)	
H8	0.6667	0.3599	−0.0434	0.066*	
C9	0.5673 (8)	0.6720 (3)	−0.4120 (4)	0.0628 (15)	
H9A	0.6335	0.6316	−0.4551	0.094*	
H9B	0.5859	0.7339	−0.4351	0.094*	
H9C	0.4272	0.6582	−0.4392	0.094*	

### Atomic displacement parameters (Å^2^)

**Table:**

	*U*^11^	*U*^22^	*U*^33^	*U*^12^	*U*^13^	*U*^23^
S1	0.0790 (10)	0.0454 (7)	0.0340 (6)	0.0035 (7)	0.0154 (6)	0.0006 (5)
O1	0.100 (3)	0.0310 (16)	0.0406 (17)	−0.0114 (18)	0.0107 (17)	−0.0004 (14)
O2	0.092 (3)	0.054 (2)	0.0415 (19)	−0.0039 (19)	0.0147 (17)	0.0101 (15)
N1	0.055 (3)	0.041 (2)	0.0303 (19)	0.0000 (18)	0.0133 (17)	−0.0019 (16)
N2	0.068 (3)	0.038 (2)	0.032 (2)	0.000 (2)	0.0133 (18)	−0.0004 (16)
N3	0.098 (4)	0.039 (2)	0.032 (2)	−0.001 (2)	0.012 (2)	0.0005 (16)
C1	0.053 (3)	0.037 (2)	0.036 (2)	0.003 (2)	0.012 (2)	0.005 (2)
C2	0.056 (4)	0.035 (2)	0.042 (2)	0.000 (2)	0.016 (2)	0.0029 (19)
C3	0.046 (3)	0.036 (3)	0.032 (2)	0.004 (2)	0.011 (2)	0.0028 (18)
C4	0.043 (3)	0.038 (3)	0.040 (2)	0.003 (2)	0.012 (2)	−0.001 (2)
C5	0.056 (3)	0.042 (3)	0.035 (2)	0.001 (2)	0.014 (2)	0.008 (2)
C6	0.069 (4)	0.061 (3)	0.033 (3)	−0.004 (3)	0.010 (2)	−0.006 (2)
C7	0.087 (4)	0.045 (3)	0.049 (3)	−0.006 (3)	0.013 (3)	−0.011 (2)
C8	0.074 (4)	0.043 (3)	0.045 (3)	−0.003 (3)	0.013 (2)	−0.001 (2)
C9	0.063 (4)	0.074 (3)	0.047 (3)	0.004 (3)	0.011 (2)	0.017 (2)

### Geometric parameters (Å, °)

**Table:**

S1—C1	1.688 (4)	C2—H2A	0.9300
O1—C4	1.358 (4)	C3—C4	1.375 (5)
O1—H1	0.8200	C3—C8	1.396 (5)
O2—C5	1.357 (5)	C4—C5	1.397 (5)
O2—C9	1.426 (5)	C5—C6	1.360 (6)
N1—C2	1.271 (5)	C6—C7	1.382 (6)
N1—N2	1.367 (4)	C6—H6	0.9300
N2—C1	1.342 (5)	C7—C8	1.365 (6)
N2—H2	0.8600	C7—H7	0.9300
N3—C1	1.315 (5)	C8—H8	0.9300
N3—H3A	0.8600	C9—H9A	0.9600
N3—H3B	0.8600	C9—H9B	0.9600
C2—C3	1.460 (5)	C9—H9C	0.9600
			
C1···C9^i^	3.425 (7)	C2···C4^i^	3.445 (7)
			
C4—O1—H1	109.5	C3—C4—C5	120.8 (4)
C5—O2—C9	118.7 (3)	O2—C5—C6	126.7 (4)
C2—N1—N2	116.0 (3)	O2—C5—C4	113.7 (4)
C1—N2—N1	119.2 (3)	C6—C5—C4	119.5 (4)
C1—N2—H2	120.4	C5—C6—C7	120.1 (4)
N1—N2—H2	120.4	C5—C6—H6	119.9
C1—N3—H3A	120.0	C7—C6—H6	119.9
C1—N3—H3B	120.0	C8—C7—C6	120.8 (4)
H3A—N3—H3B	120.0	C8—C7—H7	119.6
N3—C1—N2	116.3 (4)	C6—C7—H7	119.6
N3—C1—S1	123.5 (3)	C7—C8—C3	120.0 (4)
N2—C1—S1	120.2 (3)	C7—C8—H8	120.0
N1—C2—C3	119.8 (4)	C3—C8—H8	120.0
N1—C2—H2A	120.1	O2—C9—H9A	109.5
C3—C2—H2A	120.1	O2—C9—H9B	109.5
C4—C3—C8	118.8 (4)	H9A—C9—H9B	109.5
C4—C3—C2	119.7 (4)	O2—C9—H9C	109.5
C8—C3—C2	121.4 (4)	H9A—C9—H9C	109.5
O1—C4—C3	119.8 (4)	H9B—C9—H9C	109.5
O1—C4—C5	119.4 (4)		
			
C2—N1—N2—C1	−178.4 (4)	C9—O2—C5—C4	178.9 (4)
N1—N2—C1—N3	2.5 (6)	O1—C4—C5—O2	0.0 (7)
N1—N2—C1—S1	−177.5 (3)	C3—C4—C5—O2	179.1 (4)
N2—N1—C2—C3	−177.3 (4)	O1—C4—C5—C6	179.6 (4)
N1—C2—C3—C4	−174.4 (4)	C3—C4—C5—C6	−1.3 (7)
N1—C2—C3—C8	7.7 (7)	O2—C5—C6—C7	179.7 (5)
C8—C3—C4—O1	−179.3 (4)	C4—C5—C6—C7	0.2 (8)
C2—C3—C4—O1	2.8 (7)	C5—C6—C7—C8	0.5 (9)
C8—C3—C4—C5	1.7 (7)	C6—C7—C8—C3	−0.2 (8)
C2—C3—C4—C5	−176.3 (4)	C4—C3—C8—C7	−0.9 (7)
C9—O2—C5—C6	−0.6 (7)	C2—C3—C8—C7	177.0 (5)

Symmetry codes: (i) −*x*+2, −*y*+1, −*z*.

### Hydrogen-bond geometry (Å, °)

**Table:**

*D*—H···*A*	*D*—H	H···*A*	*D*···*A*	*D*—H···*A*
O1—H1···O2	0.82	2.14	2.610 (4)	116.
N3—H3A···N1	0.86	2.23	2.592 (5)	105.
O1—H1···S1^ii^	0.82	2.69	3.290 (3)	131.
N2—H2···S1^iii^	0.86	2.62	3.470 (4)	172.
N3—H3B···O1^iv^	0.86	2.28	2.943 (4)	134.

Symmetry codes:  (ii) −*x*+2, *y*+1/2, −*z*+1/2; (iii) −*x*+2, −*y*+1, −*z*+1; (iv) −*x*+2, *y*−1/2, −*z*+1/2.
